# Application of Modelling and Simulation Approaches to Predict Pharmacokinetics of Therapeutic Monoclonal Antibodies in Pediatric Population

**DOI:** 10.3390/pharmaceutics15051552

**Published:** 2023-05-20

**Authors:** Andrew Lim, Pradeep Sharma, Oleg Stepanov, Venkatesh Pilla Reddy

**Affiliations:** 1 Clinical Pharmacology and Pharmacometrics, Biopharmaceuticals R&D, AstraZeneca, Cambridge CB2 8PA, UK; andrew.lim20@imperial.ac.uk (A.L.); oleg.stepanov@astrazeneca.com (O.S.); 2 Faculty of Medicine, Imperial College London, London SW7 2AZ, UK

**Keywords:** pharmacokinetic modelling, PBPK modelling, popPK modelling, pharmacokinetics, pediatrics, monoclonal antibodies, physiologically based pharmacokinetic modelling, population pharmacokinetics

## Abstract

Ethical regulations and limited paediatric participants are key challenges that contribute to a median delay of 6 years in paediatric mAb approval. To overcome these barriers, modelling and simulation methodologies have been adopted to design optimized paediatric clinical studies and reduce patient burden. The classical modelling approach in paediatric pharmacokinetic studies for regulatory submissions is to apply body weight-based or body surface area-based allometric scaling to adult PK parameters derived from a popPK model to inform the paediatric dosing regimen. However, this approach is limited in its ability to account for the rapidly changing physiology in paediatrics, especially in younger infants. To overcome this limitation, PBPK modelling, which accounts for the ontogeny of key physiological processes in paediatrics, is emerging as an alternative modelling strategy. While only a few mAb PBPK models have been published, PBPK modelling shows great promise demonstrating a similar prediction accuracy to popPK modelling in an Infliximab paediatric case study. To facilitate future PBPK studies, this review consolidated comprehensive data on the ontogeny of key physiological processes in paediatric mAb disposition. To conclude, this review discussed different use-cases for pop-PK and PBPK modelling and how they can complement each other to increase confidence in pharmacokinetic predictions.

## 1. Introduction

Over the past few decades, novel developments in therapeutic monoclonal antibodies (mAbs) have helped establish their place as a mainstay in the treatment of several adult diseases, with more than 110 mAbs approved by the U.S. Food and Drug Administration (FDA) or European Medicine Agency (EMA) (Table S1) [1]. While less than 40 mAbs have been approved for paediatric indications (Table S1), the utility of mAbs in the treatment of paediatric diseases has gradually gained recognition. More recently, bamlanivimab (LY-CoV555) and REGN-COV2 were used to treat SARS-CoV-2 infections in paediatrics above 12 years old [2,3]. With the introduction of the Paediatric Research Equity Act (PREA) by the FDA and grant incentives by the health authorities to encourage paediatric research [4,5,6], there have been interesting opportunities to develop mAbs for paediatric diseases.

Despite growing interest in developing mAbs for paediatric treatment, ethical regulations and limited paediatric participants remain key barriers that hinder paediatric clinical trials [7]. These key challenges contribute to a median delay of 6 years in mAb treatment being approved for paediatric indications [8]. To overcome these barriers, the pharmaceutical industry is increasingly adopting modelling and simulation methodologies to design optimized paediatric clinical studies and reduce patient burden [9,10]. For instance, pharmacokinetic data from adults can be extrapolated to the paediatric population by modelling and simulations to inform the selection of the first-in-paediatric dose for clinical trials that balance efficacy and safety [11,12,13]. Moreover, from a regulatory perspective, the utility of modelling and simulation methodologies has been acknowledged by the FDA, which highly recommends the inclusion of modelling and simulation methodologies for paediatric study plan (PSP) submissions [14]. This mini-review aims to evaluate the classical modelling approach for mAb pharmacokinetic predictions in paediatric and their relevant knowledge gaps. Correspondingly, this mini-review explores how physiologically-based pharmacokinetic (PBPK) modelling, an emerging alternative, can be advantageous in bridging these knowledge gaps.

## 2. Methodology

### 2.1. Comparison of Modelling and Simulation Methodologies Used in Pediatric mAb Development

PubMed was used to conduct a broad systematic search in the literature for the characteristic features of different modelling methodologies. Keywords included ‘population pharmacokinetics modelling’, ‘popPK modelling’, ‘physiologically based pharmacokinetic modelling’, ‘PBPK modelling’ and ‘allometric scaling’. To narrow down the search results to paediatric and monoclonal antibodies, the keywords ‘paediatric’, ‘children’, ‘infants’, ‘monoclonal antibodies’ and ‘mAbs’ were included. Additionally, the FDA drug database was used to identify the most common modelling methodologies employed for mAb development. Each Biologics License Applications (BLA) approval for existing approved paediatric mAbs was vetted to compile modelling methodologies for use in a regulatory submission.

### 2.2. Screening Age-Dependency of Physiological Parameters for Pediatric PBPK Model Development

PubMed was used to screen the significant ontogeny of physiological parameters for paediatric PBPK development in monoclonal antibodies using the keywords ‘neonates’, ‘infants’, ‘young infants’, ‘children’, ‘adolescents’, and ‘paediatric’. This was accompanied by relevant physiological parameters of interest. Reference lists of key research articles with comprehensive datasets on paediatric physiological parameters were manually screened to seek out relevant references to corroborate our dataset.

### 2.3. Analysis of Pediatric PBPK Modelling Studies

Given the limited PBPK studies published for mAbs, a broad PubMed search was performed with the keywords ‘paediatric PBPK model’ and ‘monoclonal antibodies’ or ‘mAbs’. Subsequently, to screen for a paediatric PBPK model specific to an individual mAb, the aforementioned keyword search was accompanied by existing mAbs approved for paediatric usage. Only studies with reported AUC predicted and observed serum concentration-time graphs from which AUC could be calculated were shortlisted. Serum concentration-time graphs were digitized by Graph Reader (v. 4.0, AstraZeneca, Cambridge, UK), and AUC was calculated using R (v. 4.2.2, Core Team (2022) Vienna, Austria). Microsoft^®^ Excel^®^ (v. 2208, Microsoft Office, Washington, DC, USA), which was used for graph plotting and fold change visualization.

## 3. Results and Discussion

### 3.1. Classical Modelling Approach for Pediatric Dosing Regimen

Modelling and simulation approaches to extrapolate the first-in-paediatric dose ranged from allometric scaling based on body size to more complex physiologically-based pharmacokinetic (PBPK) and population-pharmacokinetic (pop-PK) modelling [15,16]. The strengths and limitations of these approaches are summarized in Table 1.

The classical approach that pharmacokinetic studies used for regulatory submissions to FDA and EMA were to apply both the body weight-based or body surface area (BSA)-based allometric scaling to adult PK parameters derived from the popPK model for the prediction of PK in children [16,17]. Out of the 39 mAbs currently approved for paediatric usage, only 10 mAbs have PBPK models for human adults or paediatrics.

Allometric scaling from adults to children based on body weight to determine the dosing regimen may be appropriate for mAb, where PK is primarily correlated with body weight and exhibits linear PK [18,19,20,21]. However, when mAb clearance is not scaled linearly with weight, a body-weight dosing approach could result in a clinically sub-optimal dose for children from a lower weight group [19,22]. Hence, in clinical practice, allometric scaling is rarely applied to paediatric dosing for any approved mAbs in isolation but is rather combined with more scientifically rigorous approaches such as population PK (popPK) modelling to determine safe yet effective mAb exposures for the paediatric population [5,13,18,19,20,23,24].

PopPK leverages mathematical models to evaluate pooled PK data from different clinical studies. Covariate information (weight, age or gender) can be integrated into popPK analysis, and this could help explain PK variability within the population [25]. The key advantages of this approach are the ability to analyse sparse data (typical for paediatric studies) and to identify and include the covariates that affect PK, facilitating paediatric dose selection [8]. For instance, body weight is a commonly included covariate in popPK modelling since it was established to significantly impact mAb PK.

Despite popPK modelling being commonly used to support the paediatric clinical trial design [26,27,28,29], its ability to capture the complexities of physiological changes in paediatrics and the impact of mAb on PK is restricted [30]. While age can be included as a sigmoidal maturation function (guided by the sum of gestational and postnatal age) in popPK modelling to explain the maturational differences between paediatrics and adults [8], it is crucial to acknowledge that children are not small adults. There is a growing body of evidence that suggests allometric scaling based on body weight cannot reflect the complex developmental processes that occur during paediatric growth, especially in younger age groups [31]. This is supported by several studies corroborating evidence that the rapidly changing physiology in young children could affect the pharmacokinetics of mAb. For instance, extracellular fluid volume decreases rapidly following birth, whereas plasma volume gradually increases, leading to a higher proportion of the total body volume available for mAb distribution [6,32]. While allometric scaling within popPK modelling can account for size differences between adults and children, it does not account for the aforementioned ontogeny of paediatric physiology [33], and this remains a key limitation of popPK modelling in paediatric mAb development. Given that the paediatric population is vulnerable to side effects from dosing errors, especially younger infants [34], it is imperative that modelling and simulation approaches account for how physiological differences between paediatrics and adults could affect the pharmacokinetics of mAbs and correspondingly drug exposure levels.

**Table 1:** Comparison of modelling approaches for mAb pharmacokinetic predictions in the paediatric population.

Methods	Allometric Scaling	Pop-PK	PBPK
**Characteristics**	Empirically derived function, predicting individual PK parameters (e.g., CL and V) based on demographic information (e.g., BW) (e.g., k = 0.75 for CL, 1.0 for V) [8]	Prediction based on retrospective analysis of pooled clinical data with the allometric and maturation function incorporated. Can predict within dose range studied or other doses and age range [31].	Based on understanding complex physiological processes to mechanistically predict PK based on the interplay between drug-specific characteristics [35].
**Main applications**	Extrapolate clinical PK information from adults to pediatric patients, typically combined with pop-PK to support the design of pediatric clinical studies [31]	Statistically driven analysis of pooled PK data from different clinical studies. Covariate analysis (age, gender, weight) can explain sources of PK variability. [31]	Leverage mechanistic mathematical models that recapitulate the physiology of humans, from neonates to adults, to assess the impact of ontogeny on mAb PK [36,37]
**Strengths**	Simple and quick with minimal resources required [8]	Analyse sparse data (typical for pediatric studies), and identify covariates that affect PK. Can integrate complex customised allometric and maturation functions [38].	Can be used for predictions with limited clinical data. Accounts for the ontogeny of physiological processes in pediatrics, especially younger infants, and its impact on mAb PK [39,40].
**Gaps**	Only captures body-size related information. Limited representation of complex physiological process such as TMDD or FcRn recycling. [31] Promising for mAbs with linear PK, which is affected by few well-understood parameters. However, less scientifically vigorous compared to pop-PK and PBPK [24].	Knowledge about the appropriate allometric and maturation functions required. Predictions limited to scaling of selected parameters within the population and doses studied [38].	Heavily reliant on biological understanding of ontogeny considering the physiological processes in pediatrics for initial model development. Reliability of data largely hinges on underlying ontogeny data [40,41].

BW, body weight; CL, clearance; FcRn, neonatal Fc receptor; PK, pharmacokinetics; PBPK, physiologically based pharmacokinetic modelling; pop-PK, population pharmacokinetic modelling; TMDD, Target-mediated drug disposition; V, distribution volume.

### 3.2. Physiologically Based Pharmacokinetic Modelling—An Emerging Alternative?

To address the limitations of popPK modelling, PBPK modelling, which is capable of accounting for the ontogeny of key physiological processes, was considered to determine the first-in-paediatric dose for clinical trials. PBPK models leverage differential equations which describe compartments by representing specific tissues linked by blood flow to recapitulate the anatomy and physiology of humans, from neonates to adults [36,37]. Subsequently, ontogeny in paediatric physiology, such as blood flow, lymph flow and biochemical processes, can be incorporated to evaluate their effects on drug exposure [26,42].

The utility of PBPK modelling studies has been established in small molecule drugs and plays a critical role in regulatory submissions to the FDA and EMA [35,39,43,44]. However, it cannot be assumed that similar success can be achieved when PBPK modelling is applied to mAbs, given that the PK of small-molecule and large-molecule drugs are inherently different (Table 2). Hence, the suitability of PBPK modelling for paediatric mAb development must be evaluated separately.

### 3.3. Ontogeny of Key Physiological Processes in Pediatric Monoclonal Antibody Disposition for Exploration in PBPK Studies

To provide perspectives on how PBPK studies could explore the age-dependency of key physiological processes in paediatric mAb disposition, we consolidated physiological data across different age groups from birth to adults (Table 3) and highlighted a few significant findings from a recent review evaluating the current understanding in this area [5,45].

Given that mAbs have a large molecular size, contributing to poor membrane permeability, their distribution is primarily restricted to the plasma and extracellular fluid [46]. Thus, plasma and extracellular fluid volume could be used to estimate the volume and distribution of mAbs. Clinical studies have observed extracellular fluid decreased rapidly from birth, especially over the first few months. Coupled with a modest increase in plasma volume from birth, the net total body volume of distribution was higher in young infants compared to adults [5].

While the extravasation of mAbs in paediatrics has yet to be quantified, the extravasation rates of other plasma proteins, such as albumin, which demonstrate similar distribution patterns and FcRn affinity, could serve as a proxy to explore the ontogeny of mAbs extravasation [47]. Studies have reported the higher extravasation of plasma proteins in neonates compared to adults [5], suggesting a similar trend in mAbs. The possible mechanisms explaining the higher extravasation rate of plasma proteins could be the higher proportion of ‘leaky’ tissues (tissues where capillary permeability is higher) and higher capillary density in young infants compared to adults.

Paediatric pharmacokinetic studies [48,49,50] have also reported a higher absorption rate of therapeutic proteins in infants compared to adults. Since one major absorption pathway of mAbs is lymphatic drainage and recirculation, lymph flow rate provides an indication of mAbs absorption rate [51]. While lymph flow rates have not been quantified in human infants, they are commonly estimated to be 0.2% of the plasma flow [52], which is higher in infants compared to adults (Table 3).

**Table 2:** Comparison of pharmacokinetic attributes in small molecule drugs and monoclonal antibodies. Attributes of small molecule drugs and mAbs were consolidated from research studies [45,53,54,55,56,57].

Attributes	Small Molecules	mAbs
***Molecular weight***	<500 Da	150 kDa
***Target***	Intracellular and surface targets	Membrane proteins or soluble proteins in circulation
***Route of administration***	Oral, intravascular (IV), subcutaneous (SC), intramuscular (IM)	Parenteral (intravascular (IV), subcutaneous (SC), intramuscular (IM))
***Posology***	Short-acting: often dosed daily or multiple times a day	Long-acting with dosing intervals up to months
***Absorption***	Through passive diffusion and active transporters. Usually rapid after oral administration.	Mainly through lymphatic uptake due to their large molecular size. Slow after subcutaneous administration.
***Distribution (V_d_)***	Volume of distribution high (0.1 to 1000 L/kg)	Volume of distribution is limited. Typically limited to plasma or interstitial fluid.
***Metabolism/***	Typically eliminated by CYP, UGT, transporters, renal and biliary pathways.	Intracellular catabolism by lysosomal degradation after endocytosis
***Elimination***	Mainly via biliary and renal excretion	Mainly via target-mediated drug disposition (TMDD) but can be recycled via FcRn
***Half-life (t_1/2_)***	Short (<24 h)	Long (days or weeks)
***Clearance (CL)***	Mostly linear PK; non-linearity mainly due to the saturation of metabolic pathways	Non-linear clearance is observed at low dose levels due to TMDD; linear clearance observed at above saturable dose range
***Immunogenicity***	Typically not observed	Likely generation of antidrug antibodies (ADA) due to immunogenicity. ADA can form an immune complex with mAb, which can accelerate overall mAb clearance.
***Drug–drug interaction***	Expected and need to be investigated for CYP P450 and transporter interactions	Rarely observed with some exceptions (e.g., mAbs modulating cytokine pathway may interact with CYP3A4-mediated clearance of small molecule drugs)
***Special population***	Physiological parameters (e.g. body composition, organ size, metabolic enzyme and transporter activity, plasma protein levels) affecting ADME may differ based on demographic characteristics (age, sex, ethnicity) and on comorbidities (eg hepatic or renal impairment)	Age-dependent changes in Fc receptor for the paediatric population and pregnant population

**Table 3:** Key ontogeny physiological parameters data for exploration in PBPK studies. [5,6,41,58,59,60] *FcRn p51 and B2M abundance refers to fetal values.

Key Physiological Parameters	Age Group (Years)																
	Birth	1 month	2 months		3 months	6 months	12 months		18 months	<2		2–6	6–12	12–18		Adults (>18)	Refs
**Extracellular fluid (ECF) volume %**	45	40	32		30	29	26		23	20		19	18	18		18	[6]
**Plasma volume (mL/kg)**	40	45	45		50	50	50		55	55		50	46	43		43	[5]
**Capillary density (capillaries/mm^2^)**							223			89.04		74.94	33.5	89.04		106.7 (18–40 years old) 171 (40–65 years old)	[41]
**Leaky:Tight tissue mass ratio**	0.129						0.115					0.118	0.116	0.102		0.098	[58,59]
**Endogeneous IgG concentrations (μM)**	69.26	35.16	20.21		25.05	27.79	40.21			41.68		60.53	77	76.51			[41]
**Lymph Flow**	Lymph flow data have not been quantified in pediatrics. However, research in the literature supports that the number of lymph nodes is less in pediatrics; thus, lymph flow is scaled allometrically with an exponent of 0.75 using 3.855 mL/h/kg as a reference value in adults.																[41]
FcRn p51 abundance * (pmol/mg protein)	3.36 (3.07) *			3.11				1.70			1.72				2.25		[60]
FcRn B2M abundance * (pmol/mg protein)	58.9 (40.722) *			50.2				41.3			42.1				27.7		[60]

* Refers to fetal values for FcRn p51 and B2M abundance. p51 and B2M are 2 different subunits of FcRn.

A major elimination pathway of mAbs is the lysosomal degradation after endocytosis, and clinical evidence of the Fc neonatal receptor (FcRn) role in protecting mAbs from lysosomal degradation has emerged [52]. Approximately 66% of FcRn-bounded mAbs in the vascular endothelium were recycled back to the plasma, prolonging their half-lives [61,62,63,64]. FcRn expression has not been quantified in human infants until very recently when Barber et al. investigated the ontogeny of FcRn expression in paediatric human tissues [60]. The study covered a wide developmental age range, quantifying FcRn expression in paediatric tissues (liver, intestine, kidney and skin). They discovered for the first time a declining trend in FcRn abundance from neonatal to adult levels, confirming an earlier prediction previously made by a minimal PBPK model of IgG [65].

This newfound discovery of a higher FcRn expression in paediatrics compared to adults disputed a prior hypothesis put forth by Malik et al. which proposed that lower FcRn expression levels in paediatrics could be responsible for the increasing mAb clearance observed in young infants [5]. However, there is a caveat to this hypothesis. An elevated mAb clearance in young infants could also be due to their higher level of endogenous IgG concentration after birth, resulting in increased competition for FcRn binding between IgG and mAb. Consequently, the decrease in mAb and FcRn binding could explain the decrease in FcRn recycling of mAb and, thus, increased mAb clearance. At present, an increased mAb clearance cannot be attributed to a single variable but rather can be more accurately explained by a multitude of variables. Thus, exploring the age-dependency of different physiological processes responsible for mAb elimination could provide further insights into this area.

### 3.4. Perspectives on Existing Pediatric PBPK Models for Monoclonal Antibodies

An analysis of paediatric PBPK modelling studies for monoclonal antibodies was performed to evaluate the prediction accuracy of existing PBPK models. However, paediatric PBPK studies for mAbs are still limited [15,41,65], despite the increasing appreciation of its utility in accounting for how the ontogeny of paediatric physiology could affect mAb PK [66,67]. At present, there are only seven paediatric PBPK models published [40], and these paediatric PBPK studies are limited to less than 10 mAbs relative to the 39 mAbs currently approved for paediatric usage. This is possible because the dearth of knowledge regarding age-related changes in mAb PK and the ontogeny of paediatric physiology data has hindered PBPK model development which requires rich physiological knowledge [15,41,68].

**Figure 1:**
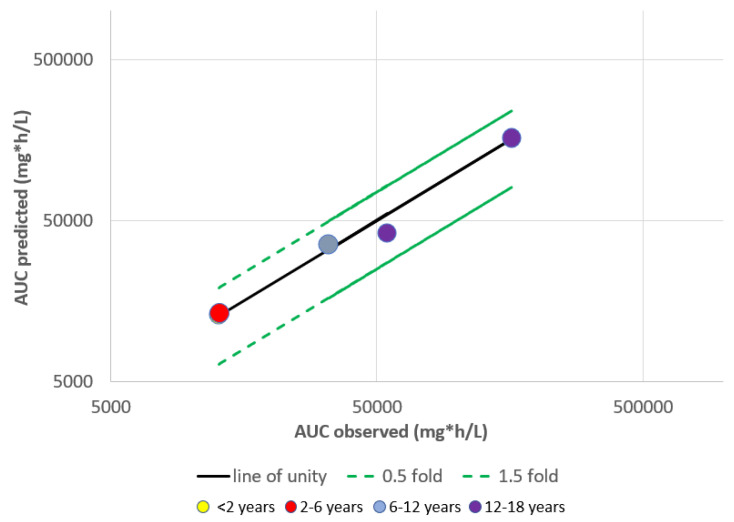
Evaluation of prediction accuracy of pediatric PBPK studies for Infliximab. Prediction accuracy of PBPK models across all pediatric age group fall within 0.5–1.5 fold change. h denotes time (in hours). Yellow data point is behind red data point as their values overlap.

Infliximab is one of the more well-studied monoclonal antibodies in terms of PBPK modelling. Pan et al. constructed an Infliximab PBPK model for full-term neonates to adolescents [41]. Using this PBPK study as an example, we evaluated how PBPK prediction accuracy varied across different paediatric age groups. The area under the curve (AUC) predicted; the over-observed ratio was used as a measure of prediction accuracy. In this study, PBPK modelling across all paediatric age groups had a prediction accuracy that fell within 0.5–1.5-fold change (Figure 1), suggesting that it is reasonably accurate. In the same study, allometric scaling using popPK parameters also found that predicted mean clearance values for infliximab fell within two-fold of the observed data [41]. These findings were also supported by Malik et al., who reported that PBPK models for infliximab achieved predictions within two-fold of the observed concentrations 66.7% of the time for children above 4 years old [19,24]. Additionally, when multiple adult popPK models were allometrically scaled for Infliximab, the poorest model still fell below the two-fold error threshold [24]. Taken together, the PBPK model for Infliximab showed comparable prediction accuracy to the classical approach of applying allometric scaling to popPK parameters.

## 4. Future Directions

**Figure 2:**
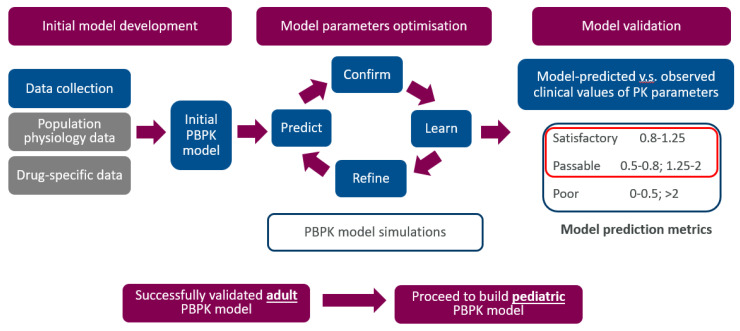
General workflow of PBPK model development based on Infliximab case study by Pan et al. Initial model development involves a ‘bottom-up’ approach to build a preliminary mAB PBPK model using drug-specific preclinical and clinical data (e.g., binding to FcRn) and systems parameters (e.g., proteomics of target expression) to inform the population model. Model is then tested to predict observed clinical data in various clinical scenarios (e.g., single ascending dose) and, if needed, optimised to capture reported clinical data in healthy adult and diseased populations. Validated adult PBPK model of mAB is then applied to predict PK in paediatrics using a population model adapted with ontogeny of physiological parameters and demography in paediatric population.

While there has been considerable progress in PBPK modelling for Infliximab, the same PBPK model does not guarantee similar success for other mAbs and disease states. Moving forward, PBPK studies should expand their scope to include the other mAbs approved for paediatric usage, subject to clinical data availability for the PBPK model development. The general workflow of PBPK model development based on the aforementioned Infliximab case study is described in Figure 2, which can be ‘adapted’ for other mAbs paediatrics PBPK model development [41]. Another aspect that future PBPK studies could explore is to account for the differences in the target receptor expression across different disease states [69], given that target-mediated drug disposition remains one of the key elimination pathways for mAbs.

However, one of the biggest challenges for PBPK modelling is that ontogeny data are still emerging, and assumptions are often made to compensate for the lack of ontogeny data. For instance, the lack of FcRn abundance in paediatrics requires the study above to assume a correlation between FcRn and IgG to study the trajectory of IgG half-lives in paediatrics. To increase the confidence of existing PBPK models, these assumptions require further validation with clinical data. Yet, there is an inherent limitation with collecting paediatric clinical data for ethical reasons and a limited sample size. Hence, PBPK studies should strive to leverage in vitro-in vivo extrapolation (IVIVE) data, which are more easily accessible, and develop optimal practices to minimize compromising prediction accuracy. With IVIVE, a preliminary exploration into the ontogeny of physiological processes could provide hints of clinically significant ontogeny for future exploration in paediatrics to reduce patient burden in clinical studies. IVIVE techniques for mAbs have made considerable progress and allow the correlation between in vivo mAb clearance and FcRn binding affinity, as well as FcRn-dependent transcytosis, to be explored [70,71]. As paediatric clinical data remain scarce, refining IVIVE techniques could be a solution that allows the translation of in vitro assay data into reliable in vivo extrapolations.

## 5. PBPK Potential Role in Regulatory Submissions for Monoclonal Antibodies

Generally, popPK modelling has been used extensively in regulatory submissions to FDA and EMA for mAb approval, and they have proved useful in explaining sources of variability in clinical PK data for mAbs. However, very few popPK analyses include neonatal and infant mAb PK data, with most paediatric popPK studies recruiting older children and adolescents above 6 years of age [8]. Additionally, studies have reported that the approach of applying size-based allometric scaling to popPK models typically works well until a lower age group is reached [45]. Thus, it remains to be evaluated if the current mAb dosing approach is safe and effective for younger infants, especially since significant physiological changes occur between birth and 2 years of age [31]. In this case, PBPK models could be valuable in accounting for the ontogeny of paediatric physiology in younger infants and increase the confidence of PK predictions of popPK modelling [8].

While PBPK models show much promise, there are some limitations to consider. For instance, the reliability of a paediatric PBPK model hinges largely on underlying ontogeny data. In cases where ontogeny data are lacking, pragmatic attempts, such as drawing parallels between PK data of endogenous and therapeutic proteins to compensate for the lack of FcRn ontogeny data, were made [41,65]. However, this approach came with residual uncertainty, given that it was based on assumptions [65]. Additionally, they required large amounts of detailed data for development and validation, contributing to a lengthy and arduous process; hence, PBPK models were less commonly utilised compared to empirical population PK models in paediatric research [5,68,72,73,74,75].

Ultimately, the type of modelling approach to use should be based on the specific research question. For instance, mAbs with a high target burden, such as trastuzumab, often exhibit non-linear PK due to TMDD. However, there was no reliable method to scale the TMDD of a non-linear popPK model by allometry [24]. In this case, PBPK’s modelling capability, when accounting for TMDD, would be highly desired [31].

It is also crucial to bear in mind that modelling approaches are not mutually exclusive and that allometric scaling, popPK and PBPK can be used synergistically together. As covered previously, allometric scaling combined with popPK modelling is the classical approach in regulatory submissions for mAb approval to the FDA and EMA. However, allometry has also been combined with PBPK modelling for therapeutic proteins in commercially available software SimCYP (SimCYP Ltd., a Certara company, Sheffield, UK)) [76]. For instance, blood flows and organ volumes for paediatrics are scaled allometrically from adult reference values. Additionally, allometric scaling can be particularly useful when ontogeny data are lacking. For instance, lymph flow data in paediatric humans has yet to be quantified, but research in the literature supports the fact that the number of lymph nodes decreases during childhood [41]. Thus, PBPK studies have explored the scaling lymph flow allometrically with an exponent of 0.75 using 3.855 mL/h/kg as a reference value in adults [41]. Acknowledging there may be residual uncertainty that arises from this approach, it is a pragmatic approach allowing the exploration of hypotheses in PBPK studies. Once future clinical data emerge to bridge the lack of existing ontogeny data, the PBPK model could be further refined to improve prediction accuracy.

## 6. Conclusions

To conclude, popPK modelling typically works well for older children above 6 years of age. However, the lack of popPK studies in neonates and younger infants does raise questions if this remains the gold-standard approach for younger infants and neonates. Especially when significant physiological changes occur in young infants, leveraging on PBPK modelling, which is a mechanistic bottom-up approach that accounts for physiological changes in young infants and could prove to be a promising tool in the arsenal of modelling and simulation methodologies. While PBPK modelling for paediatric mAbs is still nascent in its developments, the initial success in the above-discussed Infliximab case study demonstrates that it is promising in serving as a complement to the classical pop-PK modelling approach, increasing confidence in its predictions. When answering different research questions, pop-PK or PBPK have the edge over the other, and the key is to identify when to apply these modelling methodologies. Ultimately, where we draw the line between the different methodologies could constantly evolve as we deepen our understanding of mAb pharmacokinetics in paediatrics. Hence, dictating a specific methodology to be used for certain age brackets could be a premature conclusion as the drug properties in relation to systems parameters in the population, including age range, all need to be considered when answering a specific research question.

## Data Availability

No new data were created or analysed in this study. Data sharing is not applicable to this article.

## Supplementary Materials

The following supporting information can be downloaded at: https://www.mdpi.com/article/10.3390/pharmaceutics15051552/s1, Table S1: List of therapeutics monoclonal antibodies that have been approved for adult and/or paediatric usage by FDA or EMA (as of September 2022).

## Abbreviations

ADA	Antidrug antibodies
AUC	Area Under Curve
BLA	Biologics license applications
BSA	Body surface area
BW	Body weight
CL	Clearance
Cmax	Peak (maximum) plasma concentration in plasma concentration curve
ECF	Extracellular fluid
EMA	European medicine agency
FcRn	Neonatal Fc receptor
IgG	Immunoglobulin G
IM	Intramuscular
IV	Intravascular
IVIVE	In vitro-in vivo extrapolation
mAbs	Monoclonal antibodies
PBPK	Physiologically based pharmacokinetic modelling
PSP	Paediatric study plan
popPK	population pharmacokinetics
PK	Pharmacokinetic
PREA	Paediatric research equity act
SC	Subcutaneous
t_1/2_	Half life
TMDD	Target-mediated drug disposition
US FDA	U.S. Food and Drug Administration
V or V_d_	Volume of distribution
