# Supplementary material for: Application of Modelling and Simulation Approaches to Predict Pharmacokinetics of Therapeutic Monoclonal Antibodies in Pediatric Population
Source: Pharmaceutics. 2023 May 20;15(5):1552. doi: 10.3390/pharmaceutics15051552 (PMC10223156; doi:10.3390/pharmaceutics15051552)
Supplement: Supplementary file 1 [file pharmaceutics-15-01552-s001.zip › pharmaceutics-2304112-supplementary.pdf]

Table S1. List of therapeutics monoclonal antibodies that have been approved for adult and/or pediatric usage by FDA or EMA (as of Sep 2022).

Therapeutic mAbs approved for adult usage by EMA or FDA					
mAbs approved for pediatric usage		*mAbs approved for adult usage only*			
Rituximab	Blinatumomab	Abciximab	Idarucizumab	Ibalizumab, ibalizumab-uiyk	Anifrolumab, anifrolumab-fnia
Basiliximab	Evolocumab	Trastuzumab	Necitumumab	Moxetumomab pasudotox, moxetumomab pasudotox-tdfk	Loncastuximab tesirine, loncastuximab tesirine-lpyl
Infliximab	Dinutuximab	Ibritumomab tiuxetan	Alirocumab	Caplacizumab, caplacizumab-yhdp	Margetuximab-cmkb
Adalimumab	Secukinumab	Cetuximab	Daratumumab	Risankizumab, risankizumab-rzaa	Aducanumab, aducanumab-avwa
Eculizumab	Obiltoxaximab	Bevacizumab	Elotuzumab	Polatuzumab vedotin, polatuzumab vedotin-piiq	Regdanvimab
Ustekinumab	Avelumab	Natalizumab	Reslizumab	Romosozumab, romosozumab-aqqg	Tisotumab vedotin, tisotumab vedotin-tftv
Canakinumab	Emicizumab	Panitumumab	Bezlotoxumab	Brolucizumab, brolucizumab-dbll	Tebentafusp, tebentafusp-tebn
Golimumab	Benralizumab	Ranibizumab	Atezolizumab	Enfortumab vedotin, enfortumab vedotin-ejfv	Faricimab, faricimab-svoa
Belimumab	Burosumab, burosumab-twza	Certolizumab pegol	Brodalumab	[fam-]trastuzumab deruxtecan, fam-trastuzumab deruxtecan-nxki	Sutimlimab, sutimlimab-jome
Mepolizumab	Lanadelumab, lanadelumab-flyo	Ofatumumab	Inotuzumab ozogamicin	Isatuximab, isatuximab-irfc	Tixagevimab, cilgavimab
	Ravulizumab, ravulizumab-cwvz	Tocilizumab	Guselkumab	Belantamab mafodotin, belantamab mafodotin-blmf	Bimekizumab

Therapeutic mAbs approved for adult usage by EMA or FDA					
mAbs approved for pediatric usage		***mAbs approved for adult usage only***			
Ixekizumab	Emapalumab, emapalumab-lzsg	Denosumab	Sarilumab	Sacituzumab govitecan; sacituzumab govitecan-hziy	Casirivimab + imdevimab
Dupilumab	Evinacumab	Brentuximab vedotin	Ocrelizumab	Tafasitamab, tafasitamab-cxix	Mosunetuzumab
Gemtuzumab ozogamicin	Atoltivimab, maftivimab, and odesivimab-ebgn	Pertuzumab	Erenumab, erenumab-aooe	Satralizumab, satralizumab-mwge	Teclistamab
Crizanlizumab; crizanlizumab-tmca	Naxitamab-gqgk	Ado-trastuzumab emtansine	Galcanezumab, galcanezumab-gnlm	Eptinezumab, eptinezumab-jjmr	Spesolimab
Omalizumab	Ansuvimab-zykl	Obinutuzumab	Mogamulizumab, mogamulizumab-kpkc	Inebilizumab, inebilizumab-cdon	
Raxibacumab	Sotrovimab	Siltuximab	Tildrakizumab; tildrakizumab-asmn	Teprotumumab, teprotumumab-trbw	
Nivolumab	Tezepelumab, tezepelumab-ekko	Ramucirumab	Fremanezumab, fremanezumab-vfrm	Dostarlimab, dostarlimab-gxly	
Pembrolizumab	Relatlimab	Vedolizumab	Cemiplimab, cemiplimab-rwlc	Amivantamab, amivantamab-vmjw	
Ipilimumab		Alemtuzumab		Tralokinumab, tralokinumab-ldrm	
